# Cortical Motor Circuits after Piano Training in Adulthood: Neurophysiologic Evidence

**DOI:** 10.1371/journal.pone.0157526

**Published:** 2016-06-16

**Authors:** Elise Houdayer, Marco Cursi, Arturo Nuara, Sonia Zanini, Roberto Gatti, Giancarlo Comi, Letizia Leocani

**Affiliations:** 1 Experimental Neurophysiology Unit, Institute of Experimental Neurology (INSPE), San Raffaele Scientific Institute, Milan, Italy; 2 Laboratory of Movement Analysis, San Raffaele Scientific Institute, Milan, Italy; 3 Vita-Salute San Raffaele University, San Raffaele Scientific Institute, Milan, Italy; University of Toronto, CANADA

## Abstract

The neuronal mechanisms involved in brain plasticity after skilled motor learning are not completely understood. We aimed to study the short-term effects of keyboard training in music-naive subjects on the motor/premotor cortex activity and interhemispheric interactions, using electroencephalography and transcranial magnetic stimulation (TMS). Twelve subjects (experimental group) underwent, before and after a two week-piano training: (1) hand-motor function tests: Jamar, grip and nine-hole peg tests; (2) electroencephalography, evaluating the mu rhythm task-related desynchronization (TRD) during keyboard performance; and (3) TMS, targeting bilateral abductor pollicis brevis (APB) and abductor digiti minimi (ADM), to obtain duration and area of ipsilateral silent period (ISP) during simultaneous tonic contraction of APB and ADM. Data were compared with 13 controls who underwent twice these measurements, in a two-week interval, without undergoing piano training. Every subject in the experimental group improved keyboard performance and left-hand nine-hole peg test scores. Pre-training, ISP durations were asymmetrical, left being longer than right. Post-training, right ISP_APB_ increased, leading to symmetrical ISP_APB_. Mu TRD during motor performance became more focal and had a lesser amplitude than in pre-training, due to decreased activity over ventral premotor cortices. No such changes were evidenced in controls. We demonstrated that a 10-day piano-training was associated with balanced interhemispheric interactions both at rest and during motor activation. Piano training, in a short timeframe, may reshape local and inter-hemispheric motor cortical circuits.

## Introduction

The acquisition of fine motor skills can induce functional and structural cerebral plastic changes. Skill training, and particularly music practice (combining skilled, bilateral, hand motor sequences training with multisensory feedback), has been suggested as beneficial in neurorehabilitation and showed great outcomes in conditions affecting the motor system such as stroke or Parkinson disease [1–8].

Skill training modulates activity of the motor, parietal, prefrontal and subcortical regions depending on the specifics of the motor experience [9–11]. Skill acquisition is characterized by two learning phases: a “fast” learning stage that occurs during the first practice sessions of the task and that mainly involves the striatum and the cerebellum; and a second “slow” learning, which is delayed and represents incremental gains in performance after continued practice [12]. This second, “slow” learning, engages the motor cortex [12,13] and is characterized by synaptogenesis, de novo proteins synthesis and map reorganization within M1 [14,15]. Indeed, long-term practice induces progressive and specific modulations of the representation of the trained sequence of movements in M1 [12]. Pascual-Leone and coworkers, using transcranial magnetic stimulation (TMS), showed that a short-term piano training (5 days) in music naïve subjects [16] increased the cortical representation of the muscles specifically involved in the motor task and reduced their activation threshold. Likewise, professional sport players or musicians present an extended cortical sensorimotor representations of the limb used in their practice [17–19].

An electroencephalographic study (EEG) showed a progressive increase of the sensorimotor mu rhythm event-related desynchronization during implicit learning of an unimanual motor sequence, until subjects achieved complete explicit knowledge [20]. Then, mu event-related desynchronization declined until reaching baseline values. These findings were consistent with a Positron Emission Topography study showing increased activity in the primary sensorimotor cortex associated with improvement of reaction times during the implicit phase of unimanual training [21]. Indeed, cortical oscillations in the mu band (8–12 Hz) are strongly related to sensorimotor control, i.e. they react to contralateral movement and sensory stimuli. Mu desynchronization, which corresponds to an attenuation of the signal amplitude preceding and during voluntary movement, may be related to cortical activity related to movement preparation and execution [22–24].

Bimanual skills, as in piano practice, confer extra difficulty compared to unimanual tasks. Premotor cortex and supplementary motor area are considered key components of bimanual movements [25], and interhemispheric interactions between premotor and motor cortices would also play a fundamental role. An electroencephalographic study showed an increased task related power of the beta rhythm right after 30 min of a bimanual finger tapping training [26], showing an increased activity of the sensorimotor cortical areas. The authors showed that the task-related coherence was maximal during the early learning stage and decreased after training, showing an increase interhemispheric functional coupling between sensorimotor and premotor cortices during the acquisition of a novel bimanual skill. This interhemispheric functional coupling returned to baseline once the performance was stable. A functional magnetic resonance imaging study confirmed that the functional connectivity of the motor cortex network was modulated with practice and showed also an enhanced interregional coupling during the early stage of skill learning. Since longer-term motor training have been shown to induce synaptogenesis and cortical map reorganization it is likely that longer-term bimanual training might induce longer-lasting changes in interhemispheric interactions. Interhemispheric interactions can be studied in two ways using TMS. Using a paired-pulse paradigm: a conditioning pulse is delivered over one hemisphere and followed, 10 ms later, by a test pulse over the other hemisphere, inducing contralaterally a decreased motor evoked potential compared to test pulse alone [27]. This is the so-called interhemispheric inhibition at short-interstimulus interval (S-IHI). Another, more direct way, consists in stimulating one motor cortex during voluntary, ipsilateral maximal contraction of the targeted muscle. The TMS pulse is followed by a pause in the muscular activity, called the ipsilateral silent period (ISP) [27,28]. Although both S-IHI and ISP represent interhemispheric inhibition, they are due to different neuronal mechanisms [29]. ISP reflects a direct measure of the interhemispheric control of voluntary cortical motor output [30]. Since iSP directly depends on the activity of the motor cortex, it should be sensitive to sensorimotor cortical plastic changes due to motor learning and might represent an easy and straightforward method for monitoring such plastic changes involved in new skill learning.

Since musical training might have a great outcome in rehabilitation, more attention should be drawn on the intra and inter-hemispheric changes associated to such practice. Thus, in this study, we aimed to study short-term effects of a 10 day- piano training on the motor/premotor cortex activity (using task-related desynchronization (TRD)) and interhemispheric interactions (with ISP). The training schedule and piano sequences of increasing difficulty were derived from a typical educational scheme for first-level piano students.

## Materials and Methods

### Participants

Twenty-five right-handed (Edinburgh Handedness Inventory Scale [31]) healthy volunteers (16 female; median age: 24 years, range: 20–32 y.o.) were recruited and divided into two groups: experimental (n = 12) and control (n = 13) groups. No subject had ever played any musical instrument. Participants had no history of neurologic or psychiatric disorders, drug abuse, current use of psychoactive medications, neurosurgery or metal/electronic implants. The study conformed with World Medical Association Declaration of Helsinki (2013). Subjects gave their written informed consent before participating to the study which was approved by the San Raffaele Scientific Institute Ethics Committee.

### Experimental design

The experimental group (12 subjects, 9 female, mean age ± SD: 22.8 ± 1.27 years old) underwent neurophysiological assessments before and after 2 weeks of keyboard training. The control group (13 subjects, 7 female, 27.08 ± 2.87 years old) underwent the same assessments but did not train at all. Thus, EEG and TMS recordings were performed twice in an interval of two weeks. These recordings consisted in: (1) EEG/Electromyographic (EMG) recording during a musical motor task performed with an electronic keyboard, (2) TMS, to measure interhemispheric inhibition using ISP and (3) force/kinematic tests, to evaluate hand function.

The training lasted 2 weeks and consisted in 10 daily exercises (35 minutes) designed to learn a bimanual musical sequence made of consecutive notes/keys within the C major scale: C, D, E, F, G, F, E, D (each note/key corresponding to each finger) at a metronome frequency of 60 bpm. The right hand played on the fourth octave and the left hand played on the second octave. A piano teacher designed the training so that subjects could learn progressively the sequence. During each training session, subjects were performing the same exercises (see S1 Fig), increasing the rhythm every 3 days of training. The first 3 days, exercises were performed at the frequency of 60 bpm, then the rhythm was increased to 72 bpm and the last days the exercises were performed at a rhythm of 90 bpm. Subjects performed exercises with the right hand, then the left hand and then with both hand together. During the last 5 minutes of each training session, subjects performed the bimanual sequence that was asked to play during the EEG recordings at the frequency of 60 bpm.

### Keyboard performance

**Fig 1:**
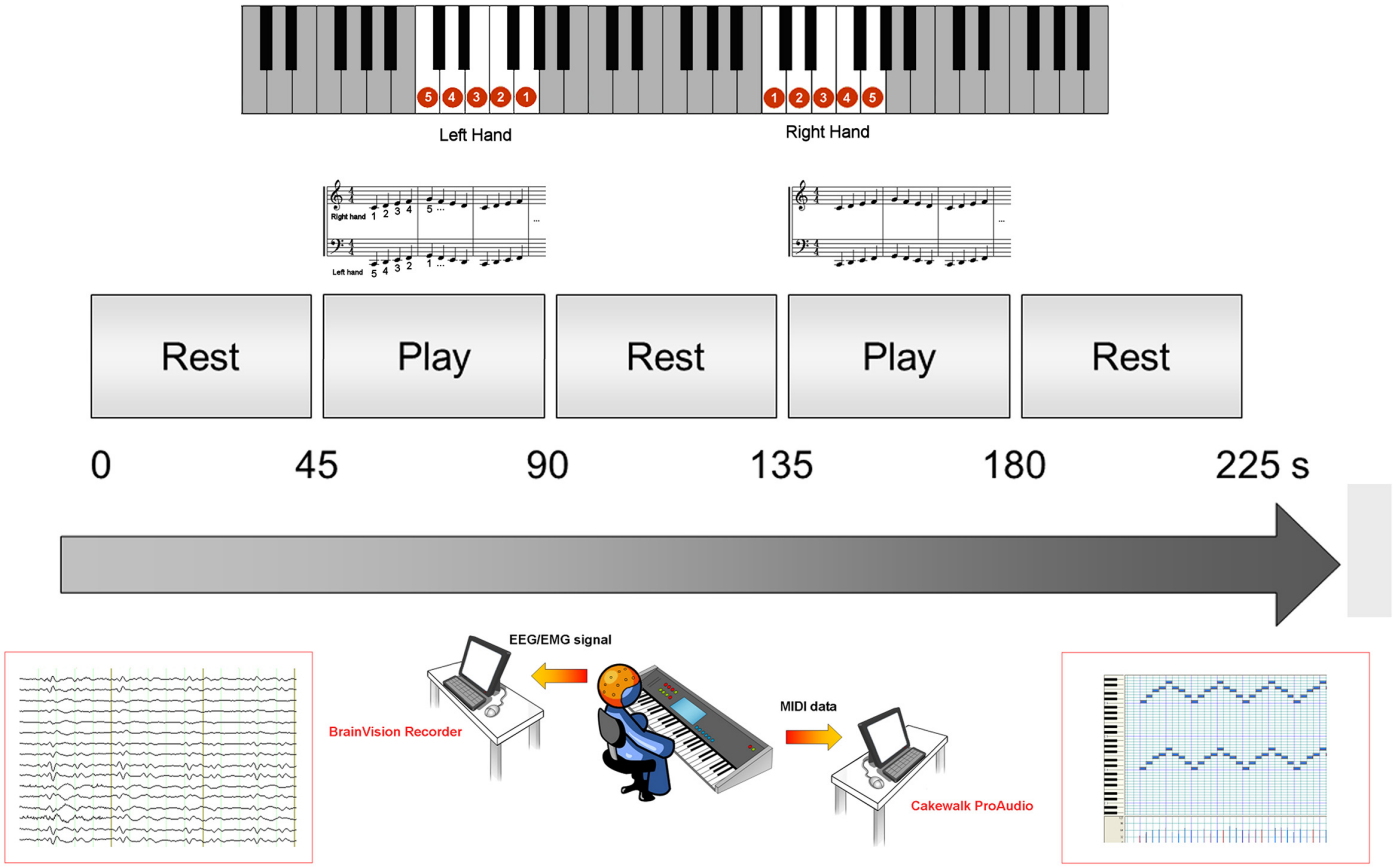
Experimental setup. EEG and EMG recording during the musical motor task performed with an electronic keyboard. Top: the blocks of motor performance are represented in terms of keyboard fingering (presented to the subjects) and standard musical notation. Center: the timing of the complete sequence is displayed. EEG and EMG signals were acquired with a PC connected to a BrainAmp system (bottom left) while MIDI data were recorded on another PC by means of a sequencing software (bottom right).

Subjects had to play the musical sequence on a keyboard TK79 (Farfisa, Potenza Picena, MC, Italy) for 60 s. The recording consisted in 2 blocks of motor performance and 3 blocks of rest, each block lasting 60 seconds (Fig 1). During rest periods, subjects kept both hands still on the keyboard. The keyboard was USB-connected to a personal computer recording each musical event, coded as Musical Instrument Digital Interface (MIDI) data. MIDI is a standard protocol to digitize musical events such as played notes, their duration, and velocity. MIDI data were recorded by the sequencing software Cakewalk ProAudio 9.03 (Cakewalk Inc. Massachusetts, USA).

### EEG/EMG recordings

EEG was continuously recorded during keyboard performance (Fig 1) from 32 Ag/AgCl electrodes fixed on an elastic cap accordingly to the 10–20 International System, referenced to the right ear lobe (A2), and ground in Oz. EMG activity from bilateral abductor pollicis brevis (APB) and abductor digiti minimi (ADM) muscles was recorded using surface Ag-AgCl electrodes in a belly-tendon montage. EMG was used to define onsets/offsets of movements, and to verify whether subjects were at rest in between the movement blocks. Signals were sampled at 5 kHz, bandpass filtered (0.3–70 Hz for the EEG and 70–499 Hz for EMG) and coded on 16 bits. Impedances were kept below 5 kΩ. EEG/EMG data were acquired using BrainAmp amplifiers and BrainVision Recorder software (Brain Products GmBH, Munich, Germany).

#### TMS recordings

TMS was delivered by a Magstim Rapid2 biphasic simulator (Magstim Company Ltd, Whitland, Dyfed, UK).

Ipsilateral silent periods were recorded using a figure-of-eight coil (Magstim 2nd generation, 70mm of external diameter) held tangentially to the scalp, the handle pointing forward rotated with an angle of 180° compared to the usual 45 latero-medial angle, in order to induce an antero-posterior current at the cortical level [32]. The coil was positioned over the best scalp location (hotspot) evoking optimal motor evoked potentials over the contralateral APB and ADM simultaneously. Fifteen stimuli were applied at an intensity of 90% of the stimulator output with 10-20s inter-stimulus intervals while subjects were performing a voluntary maximal contraction of the ipsilateral targeted muscles [29,33]. In between the TMS pulses, subjects were instructed to relax for 4–8 s. ISPs were measured bilaterally.

### Force and kinematic tests

Subjects underwent Pinch and Jamar grip tests, as well as Nine Hole Peg Test (NHPT). Pinch and Jamar tests were performed with a dynamometer (Jamar^®^, Lafayette Instrument, Lafayette, IN, USA). Subjects had to press as hard as possible with their thumb-index pinch and whole hand, respectively, three times per side, with inter-trials intervals of at least 30s. No signs of muscle fatigue were observed. NHPT score consisted on the time taken by the subject to insert every peg in the empty holes and then remove them and place them back in the shallow container, as quickly as possible [34]. The test was performed twice per side. For all the above tests, the sum of all scores, for each side, was calculated.

### Data analysis

#### Keyboard performance

MIDI data were analyzed by a home-made routine implemented in Matlab R2009a (The MathWorks, Inc. Massachusetts, USA) extracting the name of each played note, the exact time of note onset/offset (used to calculate the duration of key presses), and its velocity (MIDI parameter related to the strength of the key pressure). Using these parameters, the Matlab routine could also determine the *precision* parameter, defined as the mean of the absolute values of delays and advances with respect to the metronome clicks.

#### EEG

EEGs were analyzed using BrainVision Analyzer 2.0 (Brain Products GmBH, Munich, Germany). Data were firstly re-referenced against the average reference and low-pass filtered at 45 Hz to reduce potential high frequency noise. Gross artifacts were manually removed from the analysis, while ocular artifacts were corrected using an ICA-based correction process [35] using a value trigger algorithm to detect blinks. Blinks were thus detected on their absolute values on the EEG traces. The definitive blinks were ascertained by means of a correlation method. The ICA algorithm used was an Infomax restricted algorithm. Traces were segmented into “motor task” and “rest” periods. The “motor task” segments could be identified using the background EMG which was continuously monitored during the task to ensure complete relaxation of the subjects during rest. Then, onsets and offsets of the motor condition were marked on the EEG traces using onset and offset of background EMG. Subsequently, at least 30 2s-epochs were extracted from each segment to perform spectral analysis. Spectra were estimated by Fast Fourier Transform of Hanning-windowed EEG epochs. Spectra related to the motor condition were subtracted from those related to resting periods, in order to define, for each subject, their specific mu frequency band edges (between 8 and 12 Hz). TRD was then calculated as the difference between the mu power of the motor condition and the corresponding power of the “rest” condition, normalized to the latter and expressed as a percentage. TRD corresponds to a decrease of the mu power during the motor task, compared to rest. Maximal TRD amplitude was identified, for each condition, over each hemisphere. In order to obtain a parameter related to the extension of scalp areas showing a substantial TRD, we assigned the value “1” to each electrode showing an absolute TRD value higher than a threshold set at 30% of the TRD_max_ and the value “0” to the other electrodes. Areas were obtained by summing all the “1” values within each hemisphere, for each condition.

#### TMS

ISP durations were determined by rectifying the EMG traces before average. ISP onset was defined as the latency at which the averaged EMG activity became constantly (for at least 10ms) smaller than the averaged baseline contraction level (taken 100 ms before the stimulus to the stimulus onset) [36,37]. ISP offset was set at the first point after ISP onset at which the EMG activity regained the baseline activity. ISP duration was defined as:

$$ISP_{duration}=ISP_{offset^{}}-ISP_{onset}$$

ISP areas were normalized according to the degree of muscle contraction pre-stimulus (EMG_pre_, from -150ms to -50ms pre stimulus), in order to correct for inter-subject variability. The ISP area (in μV*ms), was calculated according to the following formula:

$$\frac{meanEMG_{pre}\times ISP_{duration}-underISP_{area}}{areaEMG_{pre}}\times 100$$

Where *meanEMG*_*pre*_ = mean EMG_pre_ activity; *underISP*_*area*_ = area under ISP [33,38].

#### Statistical methods

For each group, pre and post values of all the parameters were compared using either an ANOVA for repeated measures, or the Conover’s free distribution method, a non-parametric ANOVA based on ranks [39] depending on the normality of the data distribution, as evaluated by the Shapiro-Wilk test. Sphericity of data was tested using Mauchly’s test. In case of significance, the Greenhouse-Geisser correction was applied. For each MIDI parameter, two main factors were used: GROUP (2 levels: experimental and control) and TIME (2 levels: pre and post). For the TRD amplitude and areas, ISP duration and area, averaged baseline contraction level, and strength and kinematic data, three main factors were used: GROUP (2 levels: experimental and control), HEMISPHERE (2 levels: left or right) and TIME (2 levels: pre and post). If a significant interaction was observed between two main factors, subsequent post-hoc analyses were run using either 2 way ANOVA for repeated measures, Student’s t-tests for paired values, Student’s t-tests for independent data or their non-parametric equivalent. If a significant modulation of the TRD area was found after training, a Chi^2^ analysis was performed on each electrode to define the precise location of TRD appearance or disappearance. The presence or absence of TRD over each electrode was expressed using the “1” or “0” values assigned by our threshold calculation.

Data were considered significant when p<0.05. All statistical analyses were performed with SPSS/PC+ 13.0 (SPSS Inc, Chicago, Ill).

## Results

### Keyboard performance

**Fig 2:**
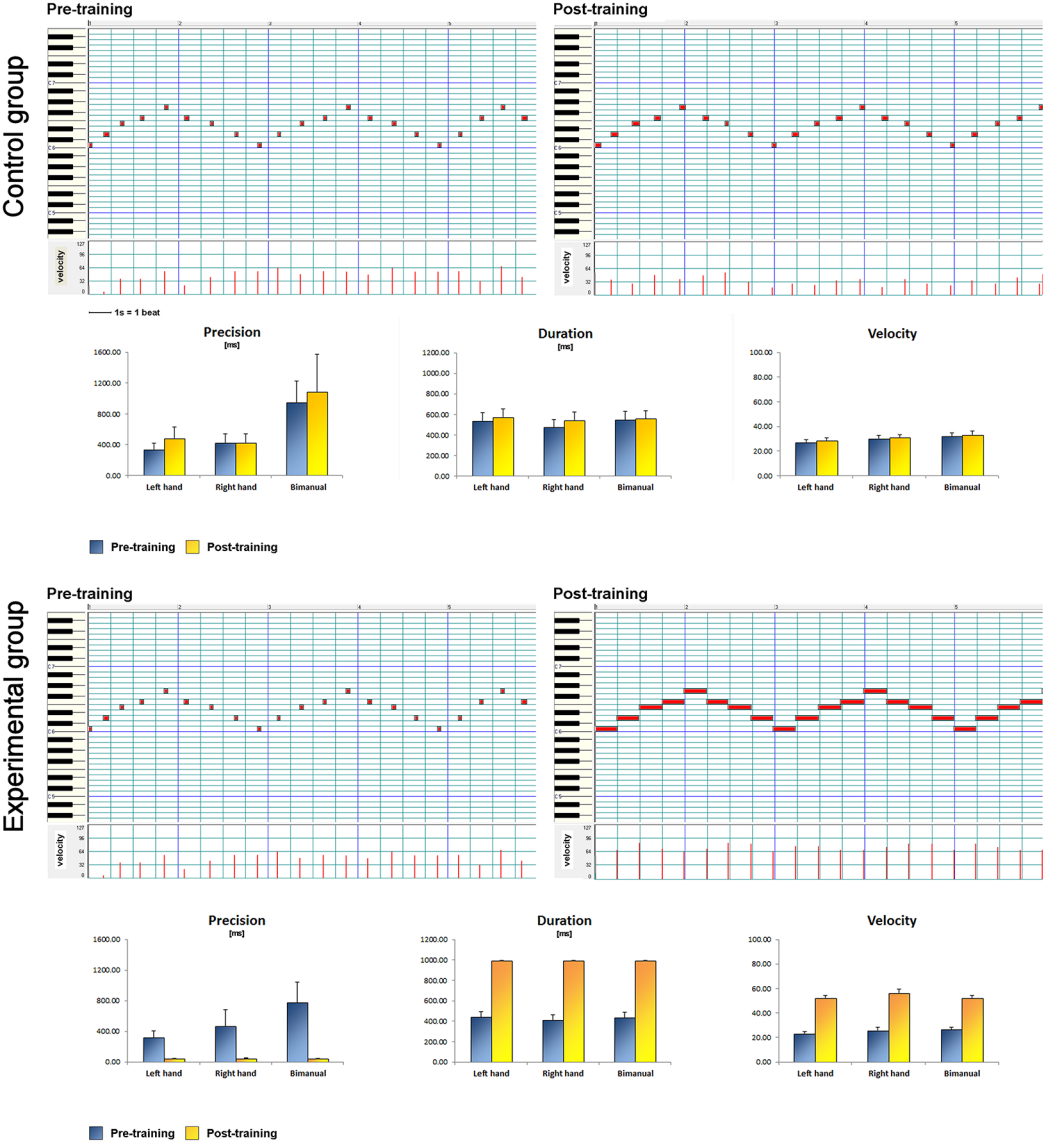
MIDI data. For each group, the piano-roll representation of the first 5 bars of the piano sequence played by a subject in the “pre” (left) and “post” (right) condition is displayed, on top of the figure. Blocks within the piano-roll show the played note (i.e. vertical position: each horizontal line represents a note, see the piano keyboard displayed on the left as a reference) and their duration (i.e. horizontal width of the block: vertical lines represent quarters within bars, the space between two vertical lines is a quarter). On the lower section of the piano-roll tables, velocities of each played note are shown. The experimental group played in a more “staccato”, soft and imprecise way before training, while a more “legato”, firm and precise style was obtained after 10 days of training. Below these graphs are represented the precision, duration and velocity as mean values + standard errors in the “pre” and “post” conditions.

All participants of the experimental group completed the full training. As reported in Table 1, every single subject of the experimental group dramatically improved his/her motor performance (Fig 2). For each MIDI parameter, the two way Conover for repeated measures showed a significant interaction between the factors TIME and GROUP (velocity: F_1, 21_ = 65.580, p<0.001; duration: F_1,21_ = 42.710, p<0.001; precision: F_1, 21_ = 13.819, p = 0.001) as well as a significant TIME effect (velocity: F_1, 21_ = 72.217, p<0.001; duration: F_1, 21_ = 55.784, p<0.001; precision: F_1, 21_ = 31.470, p<0.001). Post-hoc Wilcoxon tests showed that only the experimental group improved its performance after 2 weeks (p = 0.002 for each MIDI parameter). The performance of the control group did not show significant changes (velocity: p = 0.666; duration: p = 0.182; precision: p = 0.480).

**Table 1:** Musical performance of both groups (MIDI parameters).

	Experimental group		Control group	
	Pre	Post	Pre	Post
Velocity	25.9 ± 6.0	51.9 ± 7.4 *	32.3 ± 9.6	33.1 ± 11
Duration of key presses (ms)	374.5 ± 89.5	1018 ± 32.3 *	545.4 ± 285.9	557.3 ± 264.8
Precision	481 ± 341.8	45 ± 14.6 *	951.3 ± 960.3	1086.7 ± 1674.8

Values of velocity, duration of key presses, and precision are reported as mean ± SD before (pre) and after (post) training.

* shows "post" data significantly different from the "pre" values (p<0.05).

### EEG

A mu TRD was induced by motor performance in all but 3 subjects (1 in the control group and 2 in the experimental group). As a consequence, in order not to contaminate our data with false null values, we excluded these subjects from all the data analyses [40–43]. The whole results section (including motor performance above) is thus presented with n = 10 for the experimental group and n = 12 for the control group. EEG data are exposed in Table 2.

**Table 2:** Pre and post mu TRD values.

	Experimental group		Control group	
	Pre	Post	Pre	Post
**TRD Amplitude**				
Left hemisphere	-55.3 ± 14.7	-41.1 ± 17.8 *	-58.5 ± 11.1	-54 ± 14.3
Right hemisphere	-59.1 ± 15.4	-39.6 ± 18.3 *	-52.6 ± 13	-53.2 ± 14.2
**TRD Area**				
Left hemisphere	6 ± 0.3	4.3 ± 0.6 *	6.7 ± 1.7	6.2 ± 2.3
Right hemisphere	6.4 ± 0.3	4.9 ± 0.6 *	6.8 ± 2.1	6.3 ± 2.3

Data are expressed in means ± SD.

* shows post values significantly different from the pre values (p<0.05).

**Fig 3:**
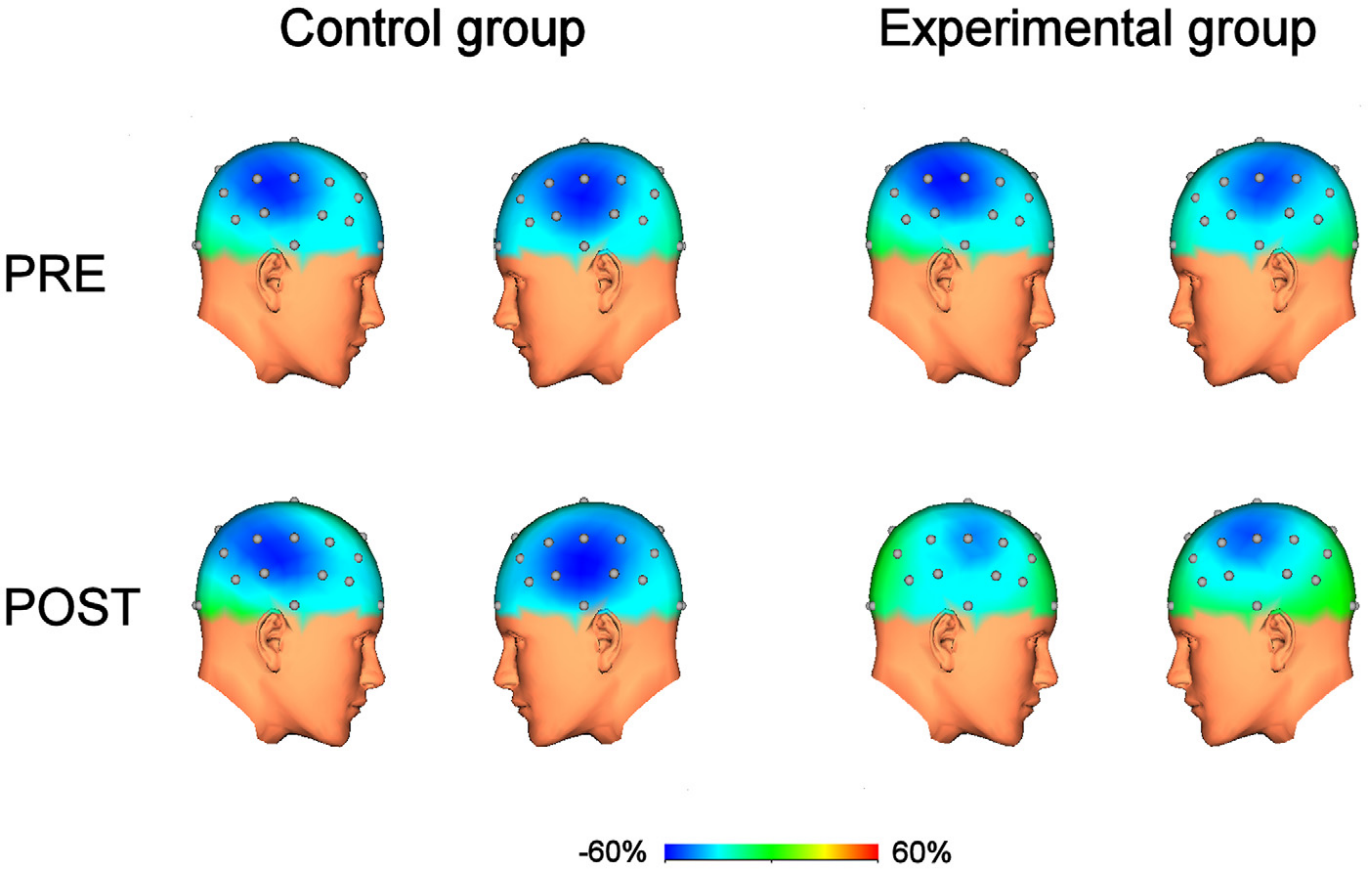
Pre and post grand-average TRD representations during motor performance in the two groups. In the control group, no significant differences were observed between the “pre” and “post” conditions. In the experimental group, motor performance-induced TRD was present bilaterally over sensorimotor areas and was significantly reduced in terms of amplitude and area after training.

Motor performance-induced TRD, present over the bilateral sensorimotor areas, was significantly modulated after training in the experimental group. Regarding TRD amplitude, the 3 way-ANOVA indicated a significant interaction between the factors GROUP and TIME (F_1, 21_ = 5.604, p = 0.029) and a significant TIME effect (F_1, 21_ = 8.881, p = 0.008). The post-hoc 2 way-ANOVAs testing the factors TIME and HEMISPHERE showed a significant TIME effect in the experimental group (F_1, 8_ = 10.599, p = 0.012) and not in the control group (F_1, 11_ = 0.252, p = 0.626). This result indicated a significant reduction of TRD maximal amplitude over both hemispheres, after training (Fig 3), in the experimental group only. Likewise, TRD areas were also decreased bilaterally after training in the experimental group (F_1, 18_ = 8.96, p = 0.009). Chi^2^ analyses revealed that TRD was significantly less represented over FC5 (p = 0.006), FC6 (p<0.001) and CP5 (p = 0.006) in post-training. In the control group, similarly to the TRD amplitude, the ANOVA for repeated values did not show any significant effects of the main factors HEMISPHERE (F_1, 11_ = 0.038, p = 0.850) or TIME (F_1, 11_ = 0.221, p = 0.648).

### TMS

**Fig 4:**
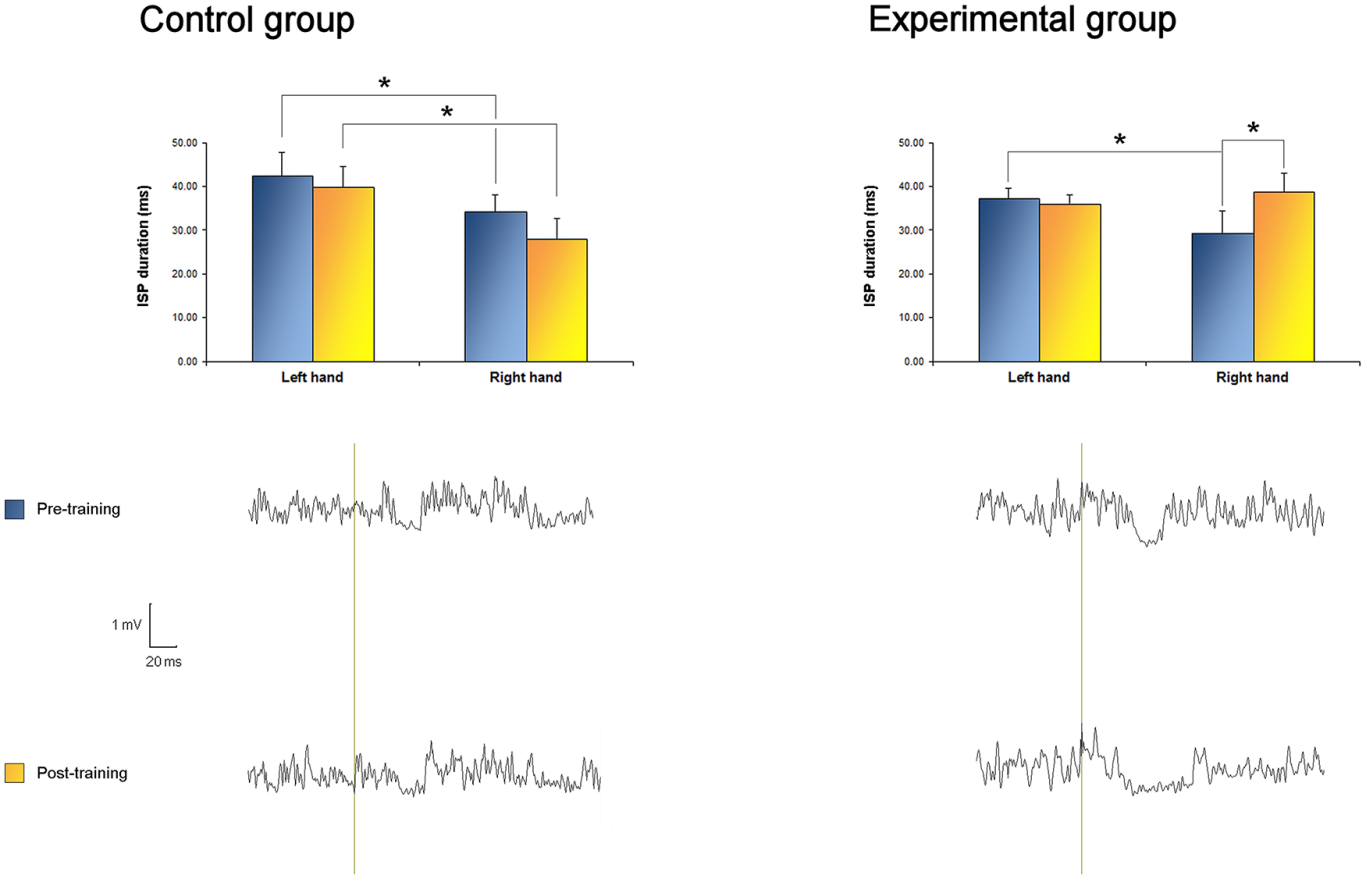
ISP_APB_ duration. Top: bilateral ISP_APB_ duration before and after training. In the control group, there was no significant effect of time. In both measurements, left ISP was larger than right. In the experimental group, right ISP_APB_ duration was significantly greater after training, leading to symmetric ISP_APB_ durations. Bottom: EMG traces recorded from the right APB muscle during right hemisphere TMS in one subject of each group, before (top) and after (bottom) training.

ISP data are presented in Table 3. Regarding the ISP_APB_ duration, the three way ANOVA showed a significant TIME x SIDE x GROUP interaction (F_1,21_ = 8.229, p = 0.009), a significant TIME x SIDE interaction (F_1,21_ = 8.229, p = 0.009), a significant SIDE x GROUP interaction (F_1,21_ = 7.395, p = 0.013), a significant TIME x GROUP interaction (F_1,21_ = 5.420, p = 0.031), and a significant SIDE effect (F_1,21_ = 26.649, p<0.001). The post-hoc two way CONOVER for repeated measures testing the factors SIDE and GROUP (pre training) showed a significant SIDE effect (F_1,21_ = 23.320, p<0.001), no GROUP effect (F_1,21_ = 1.4, p = 0.251) and no significant interaction between the two factors (F_1,21_ = 0.002, p = 0.961), thus demonstrating that, before training, left ISP_APB_ durations were greater than right ISP_APB_ duration in both groups. Post training, the 2 way ANOVA showed a significant SIDE x GROUP interaction (F_1,21_ = 18.643, p<0.001) and a significant SIDE effect (F_1,21_ = 6.711, p = 0.017). Post-hoc Wilcoxon tests indicated a significant increase of right ISP_APB_ duration after training in the experimental group (p = 0.005) but not in the control group (p = 0.205), while there were no significant changes of the left ISP_APB_ duration in the post condition (p = 0.655 in the experimental group and p = 0.386 in controls, see Fig 4). Indeed, post training, right ISP_APB_ duration were no longer different from left ISP_APB_ duration (Wilcoxon test, p = 0.060).

**Table 3:** ISP durations and areas (median ± interquartile range) in both groups in the pre and post conditions.

	APB_R	ADM_R	APB_L	ADM_L
**ISP duration**				
**Experimental group**				
**Pre**	31 ± 7.5	34.5 ± 17.8	36 ± 8.3	36 ± 22.8
**Post**	37.5 ± 14.3 *	34.5 ± 28.8	34.4 ± 13	36.5 ± 18.8
**Control group**				
**Pre**	32.5 ± 15.8	42 ± 17	40.5 ± 8	52 ± 16
**Post**	29 ± 12	44 ± 10	36.5 ± 10	47 ± 14
**ISP area**				
**Experimental group**				
**Pre**	9.9 ± 7.2	6.6 ± 12.5	13 ± 5.4	7 ± 20
**Post**	14.5 ± 7.7 *	7.7 ± 12.5	13.6 ± 5.4	9.1 ± 11.8
**Control group**				
**Pre**	15.4 ± 16.2	16.8 ± 14	19.8 ± 14.7	16.7 ± 12.9
**Post**	11.7 ± 6.9	14.2 ± 11.6	15.5 ± 18	19.2 ± 12.9

ISP durations are expressed in ms. Areas are expressed in μV x ms.

* shows post values significantly different from the pre values (p<0.05).

R: Right, L: Left.

Regarding the ISP_APB_ area, the 3 way ANOVA showed a significant SIDE effect (F_1,21_ = 4.357, p = 0.05) and a significant interaction between the factors TIME and GROUP (F_21_ = 5.015, p = 0.037). Post-hoc analyses showed a significant increase of the right ISP_APB_ area after training in the experimental group (Wilcoxon, p = 0.034) but not in controls (p = 0.534). Conversely, left ISP_APB_ areas were not changed over time (Wilcoxon, p = 0.814 in the experimental group and p = 0.074 in controls).

Regarding the ISP_ADM_ duration analysis, the 3 way ANOVA indicated a significant SIDE effect (F_1,21_ = 15.858, p = 0.001), no significant GROUP effect, no TIME effect, and no significant interactions between the main factors (p>0.05), showing that ISP_ADM_ durations were longer in the left side for both groups and did not change overtime.

The ISP_ADM_ area analysis showed not significant effects of the factors SIDE, TIME and GROUP and no interactions between the main factors (p>0.05).

The averaged baseline contraction levels of both APB and ADM muscles did not differ between groups, nor between pre and post measurements (p>0.05).

### Strength and kinematic tests

For both pinch and Jamar tests, the 3 way ANOVA for repeated measures showed a significant SIDE effect (respectively: F_1,21_ = 29.873, p<0.001, and F_1,21_ = 50.161, p<0.001) with no other significant main effects and no interactions between the main factors (p>0.05), demonstrating best performances of both groups with their right hand, and no change overtime.

Only the dexterity test (NHPT) showed significant effects of training. The 3 way ANOVA for repeated measures showed a significant TIME effect (F_1, 21_ = 6.127, p = 0.022), a significant SIDE effect (F_1, 21_ = 51.21, p<0.001) and a significant GROUP effect (F_1, 21_ = 6.15, p = 0.022). Post-hoc t-tests for paired-values revealed that left hand NHPT scores improved after training in the experimental group (mean values ± sd: pre training: 33.5 ± 4.0 s; post training: 31.6 ± 3.8 s; p = 0.005) and did not change in controls overtime (pre: 35.1 ± 3.4 s; post: 34.6 ± 2.5s; p = 0.551). Right hand performances though remained better than left hand in both groups overtime (right hand NHPT scores, experimental group: pre: 28.9 ± 3.0 s, p<0.001; post: 27.8 ± 2.5 s, p = 0.006; controls: pre: 31. 5 ± 2.5 s, p<0.001; post: 31. 2 ± 1. 6 s, p = 0.005).

## Discussion

In our findings, ten days of piano training in naïve subjects were sufficient to dramatically improve their performance, modulate inter-hemispheric communication as well as sensorimotor cortex activity.

All participants of the experimental group improved their velocity, precision and duration of key presses. Every subject played in a “staccato”, soft and imprecise manner before training; while subjects who underwent the training played in a more “legato”, firm and precise style after 10 days of training. The concurrent improvement in speed and accuracy is characteristic of the acquisition of a new skill [44]. Although in this case, the improvement in speed meant pressing each key longer than what subjects were performing before training, the concurrent improvement of the duration of key pressing, and timing accuracy led us to conclude that the experimental group had well learned the task after the 10 days of training, which was not the case for the control group.

Training was associated with a decrease in the cortical activation during motor performance, as indicated by the TRD maximal amplitude and map area. In post training, TRD was significantly less present over the electrodes FC5 and FC6, located over the premotor regions, and most presumably over the ventral premotor cortex (PMv) [45]. These results are in line with an fMRI study demonstrating a decreased activity of the bilateral PMv and dorsal premotor cortex (PMd) after music-naïve subjects had learned to play a melody on the piano keyboard with their right hand [46]. Thus, our data seem consistent with the hypothesis that the premotor cortex would be particularly involved in the early phase of motor learning in order to set up cognitive strategies and motor routines necessary to the execution of complex skilled movements [47,48], and would be less activated when the motor sequence has been learned [49]. It is noteworthy, however, that a limitation to the interpretation of our results might reside in the tendency for our two groups to show different TRD amplitude values over the right hemisphere, in the PRE condition. Although non significant, it is legitimate to hypothesize that this difference might have minimized the variation observed in POST in the control group. However, this modulation of TRD amplitude between the two groups was non significant, as well as there was no significant changes between PRE and POST values in the controls. This difference concerned only the right hemisphere, while both hemispheres showed a time effect in the experimental group. Although this difference between the two groups might thus not be responsible for our results, it would be interesting to repeat such studies in larger population in order to confirm these results. Indeed, the small sample size might represent another limitation to the interpretation of our results.

The bilateral modifications of cortical activation might have involved interhemispheric connections. The increased ISP duration and area was observed only over the APB, and not over the ADM. Since the motor sequence did not require relevant abductions of the fifth finger, it is probable that the motor control and cortical representation of the ADM might not have been modulated by the training.

Enhanced right ISP_APB_ could reflect an increased excitability of the facilitatory transcallosal fibers, and/or an increased number of active connections of these transcallosal fibers over the left, contralateral M1. Similar to primary motor cortex changes, plastic changes of interhemispheric circuits might evolve along learning and practice. Indeed, professional musicians have a larger anterior half of the corpus callosum (CC), compared to controls, especially in musicians who started to learn music earlier (Schlaug, 2001). The anterior half of CC contains fibers originating from premotor and prefrontal regions [50]. In a longitudinal MRI study conducted with children who were about to learn how to play keyboard, 15 months of training were associated with a greater development of the CC compared with an untrained control group [51]. The latter finding would favor the view that larger CC observed in musicians would be due to training. Since brain plasticity can be observed with only a few hours/days of training [16,47,49,52], we could hypothesize that 10 days of bimanual piano training were enough to modulate the callosal transmission, particularly facilitating the right-to-left interhemispheric communication (reflected by the increase of the right ISP_APB_).

The enhanced interhemispheric interaction between left and right motor/premotor areas might have, at first, participated to the setting-up of new motor commands together with the premotor cortex [47–49]. Since these increased interhemispheric interactions were still observable after 10 days of training, when the task was fully learned, this result might reflect a second-step plastic change [53], as observed in professional musicians [51,54].

It is also important to note that training improved left hand NHPT scores. NHPT is a measure of dexterity which involves fine grasping finger movements activating a large bilateral neuronal network of frontoparietal areas, especially including the PMv (for the grasping phase) and PMd (during the lifting phase) [55–58]. Precision of hand and finger movements requiring fine sensorimotor control, such as piano playing, is also dependent on the premotor cortex activity [59,57,60,58]. PMv is highly connected with sensory, motor and high-level cognitive areas and constitutes a key component of the cortical circuit involved in the sensorimotor transformations responsible for fine, visuoguided hand movements [56,59,61,62]. Our TMS data showed that motor training reinforced the right-to-left interhemispheric connections, which might have facilitated the communication between the bilateral PMv needed for the cortical motor control of fine finger movements involved in key presses and in the NHPT. This hypothesis is reinforced by the fact that only the group undergoing motor training significantly improved in NHPT performance.

The premotor cortex is also highly connected to the ipsilateral M1. PMv projects most of its afferences on the hand representation of M1 [63]. At rest, the PMv exerts an inhibition on the ipsilateral M1, modulated by the different phases of finger movement preparation and execution [61,64,65]. This inhibition is converted into facilitation during precision movements [66]. Since PMv EEG activity appeared to be reduced post-training, it could be hypothesized that the ipsilateral premotor-motor facilitation would have been reduced by motor learning. This decreased facilitation might explain, at least in part, the reduction of TRD amplitude observed over the primary sensorimotor cortex during motor performance.

## Conclusions

Our results showed how a bimanual piano training, even in a short time frame, could significantly modulate intra- as well as inter-hemispheric premotor-motor cortex functioning, most probably mediated through transcallosal connections, with plastic changes still evident three days after the end of the training, when subjects had learned the task. Our results bring additional evidence that piano training can be very efficient in inducing brain plasticity even in the early stages of learning in adults.

## Supporting Information

S1 Fig Training exercises. During each training session, subjects had to practice playing with their right hand only, then left hand only, and finally with both hands together. The exercises were decomposed so that the fingers’ involvement was brought progressively in order for the subjects to learn the whole sequence in a more pedagogic manner. The last 5 min of each session were dedicated to the training of the whole sequence. Moreover, every 3 days, the training rhythm increased from 60, to 72 and lastly 90 bpm.
(TIF)
