# Supplementary figures and images for: Cortical Motor Circuits after Piano Training in Adulthood: Neurophysiologic Evidence
Source: PLoS One. 2016 Jun 16;11(6):e0157526. doi: 10.1371/journal.pone.0157526 (PMC4911097; doi:10.1371/journal.pone.0157526)

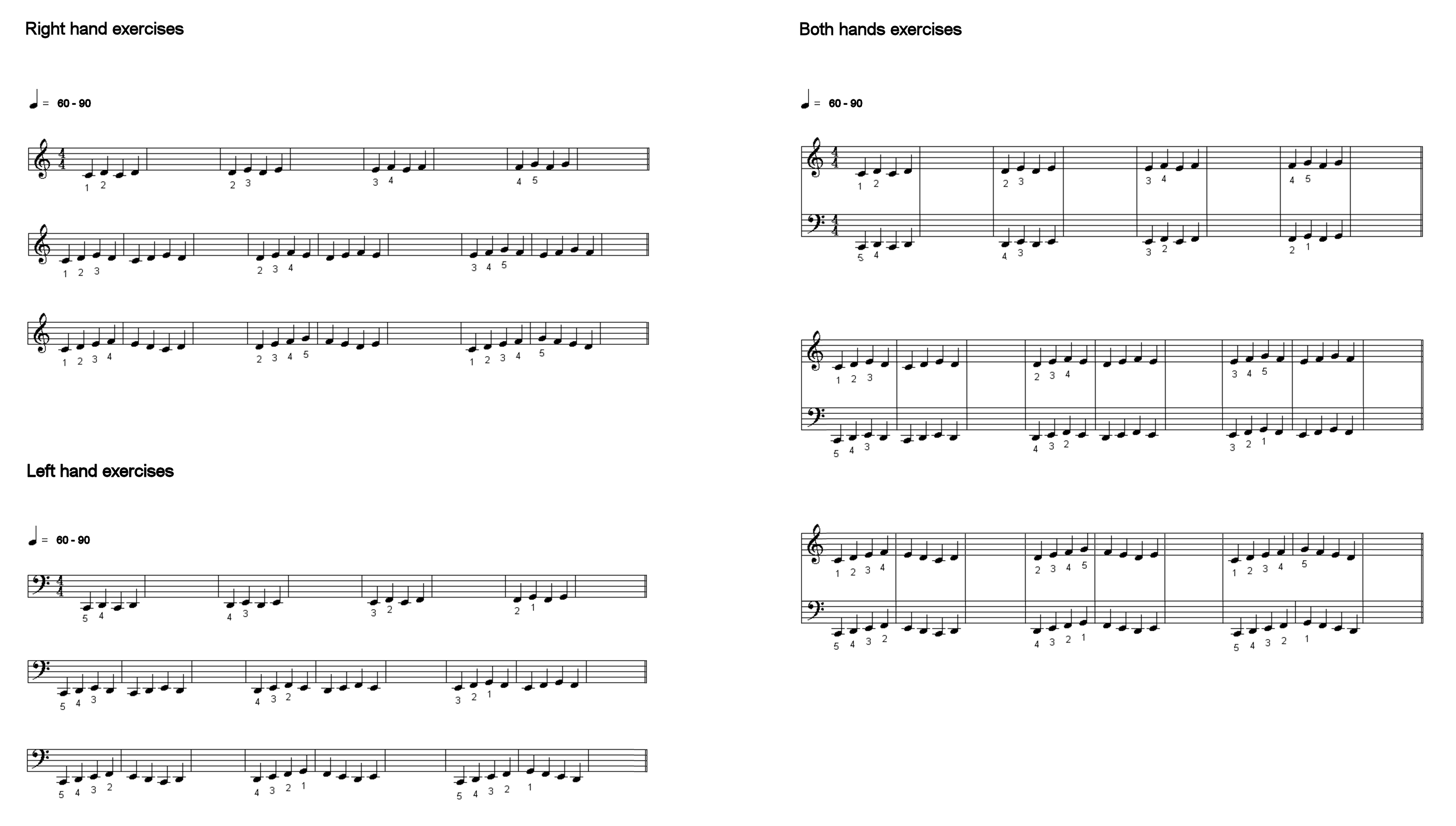

Supplement: S1 Fig — During each training session, subjects had to practice playing with their right hand only, then left hand only, and finally with both hands together. The exercises were decomposed so that the fingers’ involvement was brought progressively in order for the subjects to learn the whole sequence in a more pedagogic manner. The last 5 min of each session were dedicated to the training of the whole sequence. Moreover, every 3 days, the training rhythm increased from 60, to 72 and lastly 90 bpm. (TIF) [file pone.0157526.s001.tif]
